# Physicochemical Differences Observed in Photostability Studies of Lyophilized, Reconstituted, and Diluted Somatropin

**DOI:** 10.1007/s11095-025-03986-1

**Published:** 2025-12-16

**Authors:** Jordan D. Pritts, Uriel Ortega-Rodriguez, V. Ashutosh Rao

**Affiliations:** 1 Office of Pharmaceutical Quality Research, CDER, U.S. FDA, Silver Spring, MD USA; 2 Office of Pharmaceutical Quality Assessment III, CDER, U.S. FDA, Silver Spring, MD USA

**Keywords:** Biologics, Charge variants, Methionine oxidation, Photostability, Size exclusion chromatography

## Abstract

**Background:**

While photostability testing conditions for biologics are often based on small molecule standards, the unique characteristics of proteins necessitate a deeper understanding of appropriate testing and controls. This study examines the effects of light stress on various presentations of somatropin, a therapeutic growth hormone.

**Methods:**

Somatropin was exposed to light in lyophilized, reconstituted, and diluted forms. Quality attribute changes were analyzed using size exclusion chromatography, micro-fluidic imaging, imaged capillary isoelectric focusing, and liquid chromatography mass spectrometry (LC–MS).

**Results:**

Light stress increased high molecular weight species (HMWS), particularly in liquid formulations, as shown by size exclusion chromatography (Lyophilized + 0.4%, Reconstituted + 2.7%, Diluted + 4.7%). Micro-fluidic imaging revealed no change in particle formation. All presentations exhibited shifts in charge variants, with increases in acidic species (Lyophilized + 2.8%, Reconstituted + 7.8%, Diluted + 6.2%) and basic (Lyophilized + 0.4%, Reconstituted + 0.7%, Diluted + 0.8%) . Liquid chromatography-tandem mass spectrometry (LC–MS/MS) peptide mapping detected increased methionine oxidation in light-exposed samples, correlating with higher protein concentration (M14- Lyophilized + 2.3%, Reconstituted + 7.4%, Diluted + 2.7%, M125- Lyophilized + 2.5%, Reconstituted + 2.9%). Diluted somatropin showed higher HMWS levels but reduced methionine-125 oxidation susceptibility compared to reconstituted formulations.

**Conclusions:**

Light exposure altered product quality attributes, with more pronounced effects on liquid presentations. These findings provide insights into the distinct impacts of light exposure on different drug presentations throughout their lifecycle, highlighting the importance of tailored photostability testing for different product presentations of biologic drugs.

**Supplementary Information:**

The online version contains supplementary material available at 10.1007/s11095-025-03986-1.

## Introduction

The number of protein-based drug products has expanded in recent decades targeting a broad range of indications including cancers, inflammation, infectious diseases, and genetic disorders [1–3]. These protein therapeutics come in varying formulations and modalities including monoclonal antibodies, cytokines, growth factors, enzymes, fusion proteins, and antibody drug conjugates [4]. A large number of biotherapeutics are biotechnology derived as human, animal, plant, and microbial expression products. The added complexity due to their large size, post translational modifications (PTMs), and biological processing leads to a need for careful characterization, monitoring, and control of their production to ensure safe and effective drug products. One aspect of this characterization and subsequent control is a drug product’s photostability. Therapeutic proteins can be exposed to visible and ultraviolet (UV) light throughout a product’s lifecycle, including during the manufacturing process, dilution, and patient administration. Light exposure can lead to photo-oxidation, which results in a variety of drug product quality issues if degradation occurs within a critical site for activity that results in aggregation and loss of therapeutic efficacy [5]. Therefore, characterization and control of the photostability of a drug product is important for maintaining its quality across the product’s lifecycle [6–13].

Over 20,000 drug products have been approved by the FDA with under 900 being biologics at the start of 2024 [14]. Throughout 2024, the total number of biologic formulations had risen to 943 with 377 of those being proprietary products [4, 15]. The current recommendations for photostability testing are described by the International Conference on Harmonization of Technical Requirements for Registration of Pharmaceuticals for Human Use tripartite guideline for Stability Testing: Photostability Testing of New Drug Substances and Products Q1B (ICH Q1B) [16]. At the time that the ICH Q1B guidance on photostability was drafted in 1996 only 46 proprietary biologic drug products had been developed and approved [4, 15]. Since then, the development of biologic drug products has been steadily increasing, accounting for 32% of all FDA drug approvals in 2024 [17].

The formulation, storage, and administration of biologic therapeutics demonstrate added variability and complexity in comparison to small molecule drugs, notwithstanding the fact that many of the methodologies employed during pre-formulation and pharmaceutical development phases are derived from established knowledge pertaining to small molecules. The difference in product stability has led to a distinct approach for handling and optimization of biologic agents throughout their lifecycle, from initial formulation to patient administration. Hence, the development and optimization of improved test conditions suitable to assess the photostability of biological drug product presentations is a current need. Small molecules can be formulated into many different dosage forms (e.g., tablets, capsules, liquids, dry powders); however, the overwhelming majority (97.5%) of biologics are administered as a liquid drug product stored in either a liquid or lyophilized form with over 80% of biologics having a ‘protect from light’ designation [4]. Being administered as a liquid dosage form leads to some added mechanistic and analytical measurement complexities that are not explicitly addressed under the photostability testing conditions historically used for small molecules in accordance with ICH Q1B.

Previously, we reported on the sensitivity of marketed biotechnology derived products to light and temperature exposure [4]. In this previous data mining study of drug product labels, we observed some notable trends in temperature and light sensitivity among drug presentations, formulations, doses (single- or multi-dose), container closure systems, dosage forms, and active ingredient types [4]. Additionally, we identified some gaps in knowledge for current light and temperature stability recommendations [4]. In particular, we identified photostability recommendations in need of additional considerations for biologicals, reconstituted and diluted formulations, and liquid and lyophilized products. Biologic liquid dosage forms are commonly diluted or reconstituted in preparation for administration. In some cases, this diluted or reconstituted drug product can be stored up to 60 days [4]. However, there are no standardized recommendations on testing the photostability of drug products after they have been prepared for administration. These gaps become apparent when taken in the context of biologics as they are commonly administered as infusion liquids. The incidence of unexpected and high levels of particulate formation, aggregation, and adverse events related to product quality issues of reconstituted or diluted products has been previously reported by others [18–24].

Herein, we tested the effects of light exposure on somatropin, a therapeutic growth hormone, as a proof of principle protein-based biologic drug product. Our data collectively report the photostability of somatropin, support the development of pharmaceutically relevant testing conditions to design or control photostability, and provide insights into the photostability risks of biologics in clinically relevant scenarios.

## Materials and Methods

### Materials

Somatropin was obtained from WEP Clinical. Cell culture grade water (cat#: 25–055-CV) and phosphate-buffered saline (PBS) pH 7.4 without calcium and magnesium (cat#: 21–040-CM) were purchased from Corning Inc, Corning, NY. Acetone (cat#: A929-1), iodoacetamide (IAA) (cat#: A39271), trifluoro acetic acid (TFA) (cat#: 85,183), acetonitrile (ACN) (cat#: 85,188), and formic acid (FA) (cat#: 28,905) were obtained from Thermo Fisher Scientific, Waltham, MA. Trypsin and LysC (cat#: V5073) were obtained from Promega, Madison, WI. Cartridges (cat#: PS-MC02-C) for imaged capillary isoelectric focusing (icIEF) were purchased from Bio-techne/ProteinSimple, San Jose, CA. Pharmalytes (cat#: 17,045,601) were obtained from Cytiva, Marlborough, MA. Eight molar urea solution (cat#: 51,457) was obtained from Sigma-Aldrich, St. Louis, MO. Two mL Type 1 borosilicate glass vials (cat#: 223,683) and chlorobutyl stoppers (cat#: 224,100–072) were obtained from DWK Life Sciences, Vineland, NJ.

### Light Exposure

Samples were placed in a photostability chamber (Caron, model: 7545–11-2) with a condensate recirculator (Caron, model: CRSY-102–1). Samples were exposed to light to meet current ICH Q1B recommendations of 1.2 million lux hours of visible light and 200 W/m^2^ of UV light with light intensity maintained at 20 klux and 20 W/m^2^ respectively. Light intensity was continuously monitored for both visible and UV exposure using calibrated radiometers positioned within the photostability chamber with readings every 15 min. Temperature and humidity were maintained at 25 ± 2 °C and 65 ± 5% RH during exposure. Lyophilized samples were exposed in the manufacturer’s drug vial with the label removed. Reconstituted samples were reconstituted with the supplied diluent according to manufacturer’s directions to 5 mg/mL and exposed in the manufacturer’s vial with the label removed. Diluted samples were reconstituted similarly to above, diluted with sterile PBS to 0.5 mg/mL, and 1 mL aliquots were pipetted into 2 mL borosilicate glass vials. Control samples were prepared by wrapping samples with tinfoil and placing them on the chamber’s shelf with their corresponding light exposed versions for the duration of the experiment. These samples are referred to as “dark controls” throughout the manuscript. All samples were exposed in an upright vial orientation mimicking typical storage and handling for the drug products. Exposures and dark controls were conducted on 1 vial respectively of each product presentation. Samples were then aliquoted and prepared in triplicate for each analysis and run once to form the triplicates referred to throughout this study.

### Size Exclusion Chromatography (SEC)

Lyophilized, reconstituted, and diluted samples were aliquoted and if applicable diluted to 0.5 mg/mL prior to SEC analysis. Samples were centrifuged at 12,000 × g for 10 min at 4 °C and the supernatant was transferred to a Waters Qsert vial. Somatropin products utilized 5 µL injections onto a Waters Acquity UPLC with a BEH SEC column, 125 Å, 1.7 µm, 4.6 mm × 150 mm at 0.3 mL/min. A mobile phase of sterile PBS (pH 7.4) was used. Absorbance was monitored at 220 and 280 nm, and fluorescence was monitored with an excitation at 280 nm and an emission at 340 nm. Empower software was utilized for assessing peak area. Bar graphs and chromatograms were plotted using Graphpad Prism v10.0.3. Dark controls were observed to be similar and were combined for analysis. Statistics were performed in Graphpad utilizing a one-way ANOVA compared to the control samples.

### Micro-Flow Imaging (MFI) for Particle Analysis

300 μg of somatropin was diluted to 1 mL with sterile PBS just prior to running particle analysis on a Protein Simple micro-flow imager 5200. Particles were measured between 1–150 μm and reported as particles per mL. Baseline measurements between samples were conducted utilizing PBS until total particle counts reached below 200 counts/mL. Bar graphs showing mean ± SD were plotted using Graphpad Prism v10.0.3. Dark controls were observed to be variable between presentations and were left un-pooled. Data were analyzed utilizing a one-way ANOVA to each sample’s corresponding dark control.

### Sample Preparation for Liquid Chromatography-Tandem Mass Spectrometry (LC–MS/MS)

Twenty-five µg of somatropin was diluted to 100 µL with Milli-Q water. Samples were diluted 1:5 with 400 µL of acetone, incubated overnight at −20 °C, and centrifuged at 15,000 × g at 4 °C for 10 min. Supernatant was discarded, the pellet was washed with 1 mL of cold acetone, and centrifuged at 15,000 × g at 4 °C for 10 min. The supernatant was discarded, and the pellet was air dried. Samples were processed utilizing S-trap micro columns from ProtoFi according to manufacturer’s recommendations. Briefly, 11.5 µL of buffer 1 and 11.5 µL of Milli-Q water was added to each sample and mixed by trituration. 1 µL of buffer 2 was added, the sample was mixed, and incubated at 55 °C for 15 min. The protein was alkylated with buffer 3 by adding 1 µL of buffer 3, mixed, and incubated at room temp in the dark for 10 min resulting in dithiomethane modified cysteine residues. 2.5 µL of buffer 4 was added to each sample and vortexed. 8.1 mL of LC–MS grade methanol was added to buffer 5 and 165 µL was added to each sample and mixed. Sample was applied to the S-trap and placed in a 2 mL Eppendorf tube. Samples were washed 3 × with 150 µL of buffer 5. Samples were placed in fresh 2 mL Eppendorf tubes. Trypsin/LysC was reconstituted utilizing buffer 6 and 20 µL was added to each s-trap targeting a 1:10 wt:wt ratio of trypsin/LysC to somatropin. Samples were placed in a humidified Eppendorf thermomixer at 37 °C overnight. Samples were eluted 3 × with 40 µL each of buffer 6, 0.2% formic acid in water, and 50% acetonitrile in water.

Samples were speed vacuumed to dryness. Pellets were resuspended in 100 µL of 3% ACN/0.1% FA in water, centrifuged at 10,000 × g at 4 °C for 10 min, and 75 µL was aliquoted into a Waters Qsert vial. Samples were analyzed using a waters Xevo G2-XS Q-TOF Nano-LC/MS system utilizing a nano-EASE M/Z HSS C18 T3 column (100A, 1.8 µm, 75 µm × 100 mm). A gradient of 2%−40% ACN for 60 min, 40–90% ACN for 61–65 min, 90–2% ACN from 66–90 min was used at a flow rate of 0.5 μL/min. Both water and ACN mobile phases contained 0.1% FA and the column temperature was maintained at 45 °C. Mass spectrometer was set to sensitivity mode scanning over the range of 100 Da to 2000 Da with a scan time 0.5 s. A collision energy ramp of 15–35 V was used. A general search of PTMs was conducted by PEAKS Studio 11 from Bioinformatics Solutions Inc. Search parameters included precursor and fragment ion mass error tolerances of 0.1 Da, peptide lengths of 6–30 amino acids, and a maximum of 2 PTMs per peptide. The ion extracted chromatogram peak area for each identified PTM from MassLynx (Waters) was then utilized for relative quantitation of modified and unmodified peptides. Data are shown as mean ± SD with at least 3 replicates per sample and one-way ANOVA tests were utilized for statistics in Graphpad Prism v10.0.3. Dark controls were observed to be similar and combined for analysis.

### Charge Variant Analysis Via Imaged Capillary Isoelectric Focusing Icief

Charge variant analysis of somatropin samples was performed on a Maurice capillary electrophoresis instrument as described by the manufacturer. Briefly, 15 µg of protein was diluted in 200 µL of a 4% Pharmalyte broad range pH 3–10 ampholyte carrier solution containing 12.5 mM arginine, 12.5 mM iminodiacetic acid and 3.2 M urea. Isoelectric point markers of 4.05 and 10.17 were used. Pre-focusing was performed for 1 min at 1500 V, and focusing time was set to 6 min, 3000 V. Peak detection was obtained in fluorescence mode for 20 s. icIEF data processing was performed on Compass for iCE (Bio-Techne). The peak areas of charge variants were plotted as bar graphs using Graphpad Prism v10.0.3 and presented as the mean ± SD. Representative electropherograms were generated with Graphpad Prism v10.0.3.

## Results and Discussion

### Post Light Exposure Product Purity Assessment Via Size-exclusion Chromatography (SEC) and Micro-Flow Imaging (MFI)

SEC and MFI were utilized to detect and quantify the effects of light exposure on soluble and insoluble aggregates, respectively. Somatropin aggregates have been previously reported to induce immunogenic responses and increase the risk of development of anti-somatropin antibodies, which could result in diminished drug efficacy as observed in previous reports [25, 26]. Additionally, light exposure has been previously shown to induce aggregation and degradation of various proteins via SEC [5–7, 13, 27–31]. Previous reports primarily focused on one presentation type, either liquid or lyophilized during exposure, therefore the differences in size variants observed after light exposure and the comparison of liquid and lyophilized formulations is open for further investigation.

**Fig. 1:**
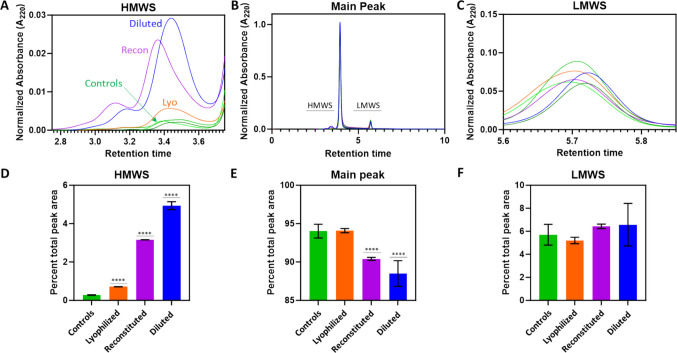
Analytical characterization of somatropin post light exposure via SEC. Representative SEC chromatograms of samples post light exposure highlighting the HMWS (**A**), full chromatogram (**B**), and LMWS (**C**) regions. Bar graphs detailing the percent total peak area of the HMWS (**D**), main peak (**E**), and LMWS (**F**) identified from the SEC chromatograms. Samples conducted in triplicate ± SD. Green – dark controls, Blue – diluted product exposed samples, Orange – Lyophilized product exposed samples, Purple – reconstituted product exposed samples. Statistics derived from a one-way ANOVA compared to the combined control samples. Statistics derived from a one-way ANOVA compared to the control sample (**** *p* ≤ 0.0001).

Hence, we used SEC which allows the relative quantitation of monomeric protein *versus* soluble high molecular weight aggregates and low molecular weight species (LMWS) that may arise from degradation of the drug product as a result of light exposure. We utilized SEC to investigate the effects of light exposure on lyophilized, reconstituted, and diluted forms of somatropin to assess protein purity (including product-related species) post exposure (Fig. 1a-c). All dark control samples were observed to be comparable and were pooled for analysis. Up to a ~ 5% increase in high molecular weight species was observed in all light exposed samples compared to their dark controls as indicated by a cluster of new peaks in the overlayed chromatograms at retention times (RTs) of ~ 3.1 and ~ 3.45 min (Fig. 1a). This effect was greater in presentations that were liquid at the time of exposure (reconstituted and diluted) (Fig. 1d). Lyophilized somatropin demonstrated the least amount of HMWS species associated with light-induced damage (i.e., ~ 0.4%) compared to reconstituted or diluted liquid forms (at ~ 3% or ~ 5%, respectively), indicated by comparable main peak and LMWS to the dark controls (Fig. 1e,f). A small (~ 0.4%) but statistically significant increase in HMWS was observed between the lyophilized and dark control samples (Fig. 1d).

**Fig. 2:**
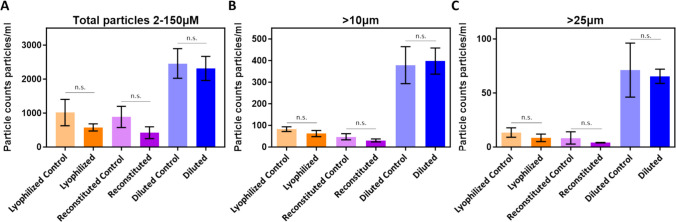
Particle analysis of somatropin post light exposure. Bar graphs detailing total particles per milliliter between 2–150 µm (**A**), above 10 µm (**B**) and above 25 µm (**C**) in size. Blue-diluted product exposed samples, Orange-Lyophilized product exposed samples, Purple-reconstituted product exposed samples. Statistics derived from a one-way ANOVA compared to each control sample. Statistics derived from a one-way ANOVA compared to the control sample (n.s. not significant).

MFI aids in identifying insoluble aggregates and subvisible particulates that may go undetected by traditional SEC techniques. Particle counts were analyzed to determine if light exposure resulted in an increase in insoluble aggregates, in accordance with USP < 788 > [32]. However, there were no differences observed in total particulate counts (Fig. 2a), in particulates > 10 µM, or > 25 µM (Fig. 2b,c) between stressed samples and their dark controls. These data are supported by a previous finding that light exposure did not affect particle formation in an IgG liquid formulation [31].

These data suggest that light exposure can affect somatropin purity by inducing HMWS in the form of soluble aggregates with greater impact seen in formulations exposed to light in their liquid forms. However, the increase in soluble aggregates (by SEC) seemed stable in all presentations with no observed exacerbation to insoluble aggregates (by MFI) in either liquid or lyophilized forms.

### Charge Variant Analysis Post Light Exposure

Therapeutic proteins can have a variety of charge profiles based on their conformation, amino acid content, and PTMs. Additionally, charge heterogeneity of protein therapeutics can be a critical quality attribute (CQA) and changes in this CQA can have implications affecting protein quality including decreased solubility, particle formation, and loss of efficacy [32, 32]. Light exposure has been previously identified as a stress condition that can modify charge profiles of proteins leading to increased acidic variants and attributed to light mediated aggregation and fragmentation products [6, 29–31]. The extent to which light can induce charge variant changes in somatropin and whether those effects are observed differently when treated on solid and liquid dosage forms was unclear. To investigate the effects of light exposure on somatropin in different presentations, analysis by icIEF was conducted.

**Fig. 3:**
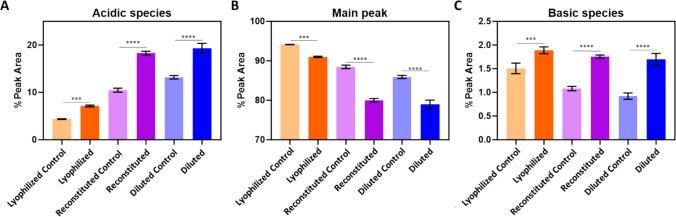
Charge variant analysis of somatropin post light exposure. Bar graphs showing the total percent peak area of the identified acidic species (**A**), main peak (**B**), and basic species (**C**) derived from icIEF chromatograms. Performed in triplicate with mean ± SD shown. Blues-diluted product exposed samples, Oranges-Lyophilized product exposed samples, Purples-reconstituted product exposed samples. Statistics derived from a one-way ANOVA compared to each control sample (*** *p* ≤ 0.001, **** *p* ≤ 0.0001).

Light exposed somatropin samples were consistently observed to have increases (e.g., up to ~ 8% for reconstituted samples Fig. 3a) in both acidic and basic variants with a corresponding decrease in their main charge variant when compared to their dark controls (Fig. 3a-c and Figure S1a-c). Liquid formulations were more sensitive to light-induced charge variant changes than lyophilized samples (Fig. 3). The dark controls were observed to be variable between presentations for this analysis, so the controls were not pooled. The variability in the dark controls was attributed to increased deamidation observed in the liquid samples from residing in their liquid form for a longer duration of time before icIEF analysis. Increased deamidation in liquid formulations was confirmed by LC–MS but not observed to be light dependent (Table S1 and Figure S2).

Together these data show that light exposure can induce changes in somatropin charge variants with increased effect on liquid formulations and acidic variants.

### PTM Analysis Post Light Exposure

Methionine, tryptophan, tyrosine, phenylalanine, cysteine, and histidine oxidation have been shown to be induced by light exposure most likely via the generation of reactive oxygen species (ROS) [5, 6, 13, 28–32]. Methionine oxidation is one example of a common PTM that has been shown to affect protein stability and function. Additionally, drug product formulation components like citrate buffer and polysorbate, as well as potential contaminant metals such as Fe have been shown to enhance these light degradation effects through their ability to act as ROS generators [7, 28]. Specific to somatropin, the disulfide bonds and tryptophan 86 of somatropin have been shown to act as ROS generators [32, 32, 32]. However, the effects of protein presentation during light exposure on methionine oxidation has not been investigated fully.

Previous studies with monoclonal antibodies (mAbs) have demonstrated protein concentration-dependent photooxidation effects, though these investigations were conducted exclusively under visible light exposure (400–800 nm) without UV components [28]. In contrast to these mAb studies, our somatropin investigation employed ICH Q1B conditions incorporating both visible light (1.2 million lux hours) and UV light (200 W/m^2^), representing a comprehensive photostability assessment that may reveal different degradation mechanisms. The mAb studies showed increased methionine oxidation with higher protein concentrations under visible light alone, suggesting the presence of unknown photosensitizers that become more concentrated with increasing protein content [28]. However, the addition of UV light in our study design may introduce direct photochemical pathways not observed in visible light-only conditions, potentially leading to different concentration-dependent relationships or alternative degradation mechanisms in the context of somatropin.

**Fig. 4:**
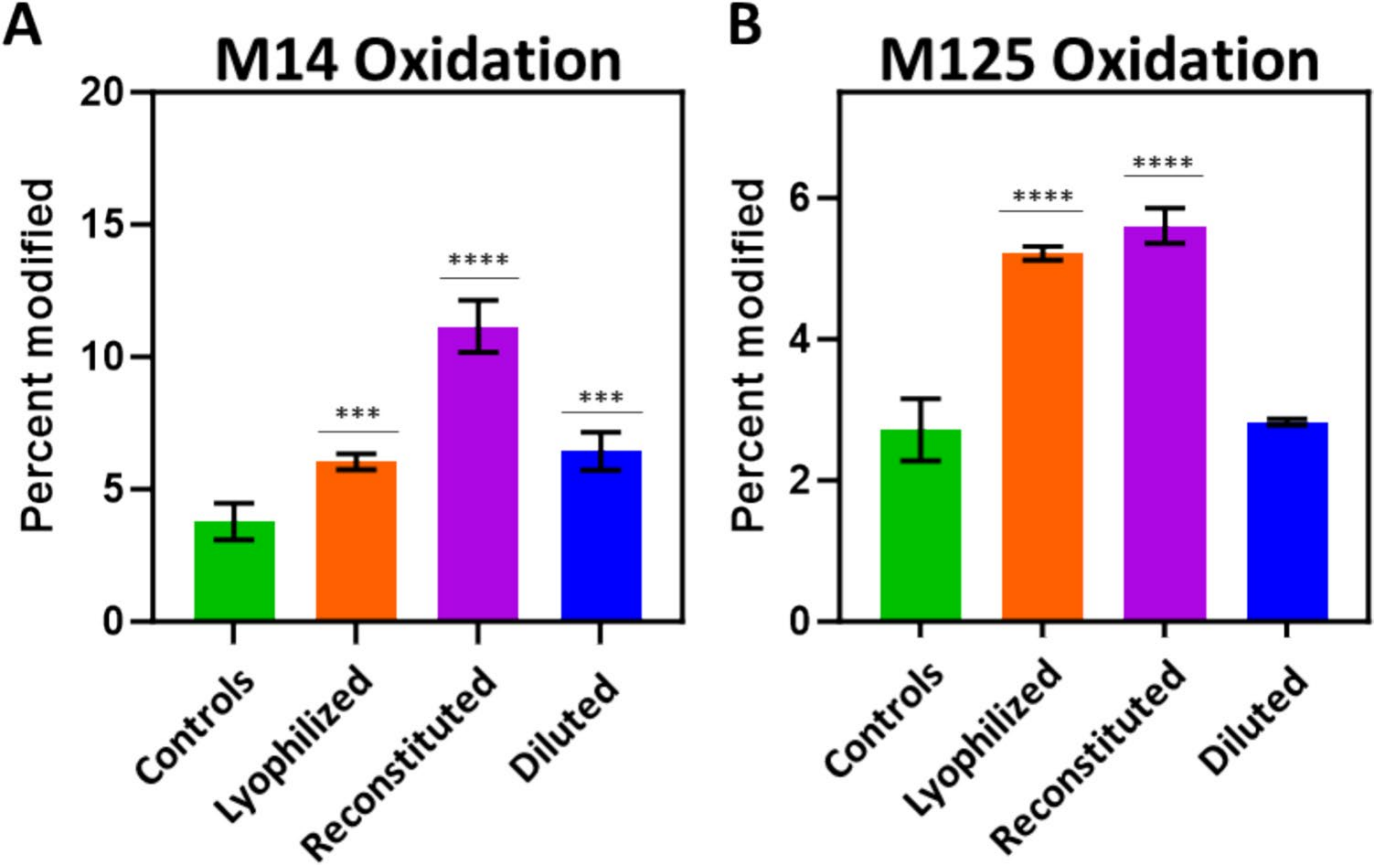
PTM analysis of somatropin post light exposure via LC–MS. Bar graphs showing the percent oxidation of methionine 14 (**A**) and methionine 125 (**B**). Green – dark controls, Blue-diluted product exposed samples, Orange-Lyophilized product exposed samples, Purple-reconstituted product exposed samples. Performed in triplicate with mean ± SD shown. Statistics derived from a one-way ANOVA compared to the control sample (*** *p* ≤ 0.001, **** *p* ≤ 0.0001).

To address this gap and compare concentration effects under combined UV/visible light conditions, somatropin was exposed to light in lyophilized, reconstituted, and diluted forms and analyzed by LC–MS/MS to determine the corresponding light induced post translational modifications that could be identified. Utilizing LC–MS/MS, methionine residues 14 and 125 were identified to have light induced oxidation (Fig. 4a,b and Figure S3a,b). Dark controls were comparable and pooled for analysis. Methionine 125 oxidation followed a trend similar to that previously identified in mAb studies under visible light-only conditions [28], where increased protein concentration led to increased methionine oxidation. In our study, this concentration-dependent effect was observed between the reconstituted formulation (5 mg/mL) and the diluted presentation (0.5 mg/mL), suggesting that concentration-dependent photooxidation mechanisms may be conserved across different protein types and light exposure conditions. However, the inclusion of UV light in our ICH Q1B conditions may have contributed to the observed effects through direct UV-induced photochemical reactions in addition to any visible light-mediated photosensitization processes. Additionally, our data shows a small but statistically significant increase in methionine 14 oxidation and no change in methionine 125 oxidation for the diluted product. The diminished overall effects of methionine oxidation upon light exposure after dilution leads to a few possible explanations. Either dilution leads to lower probability of somatropin to come into contact with radical oxygen species generated from light due to increased molecular diffusion or an unknown photosensitizer is also lowered in concentration upon dilution. Additionally, it is possible that due to dilution, there is less somatropin near the glass interface during exposure where light intensity is the highest before light scattering and diffusion. Deamidation at asparagine residues 149 and 152 were observed, but no differences between light exposed and dark control samples were distinguishable (Table S1 and Figure S2). These data are consistent with previous reports that observed methionine oxidation and deamidation present in unstressed somatropin samples [32].

Methionine residues 14 and 125 are within 15 angstroms of one another in the somatropin crystal structure (PDB: 1A22). However, they are on opposite sides of the protein. For the lyophilized product, a more significant increase in methionine oxidation was observed for methionine 125 *versus* its control sample compared to the increase observed in methionine 14. One possible explanation for this observation is that somatropin in its lyophilized state may structurally orient in a manner that leaves more of the side containing methionine 125 facing towards the glass of the vial leading to increased light exposure. This observation is in alignment with a previous study that showed near-UV exposure of bovine somatropin in its solid state resulted in photoionization of tryptophan 86 and subsequent reduction of disulfide bridges by solid state Raman spectroscopy [32]. Tryptophan 86 and the disulfide bridge between cysteines 53 and 164 are on the same side of the protein as methionine 125. Additionally, it was previously reported that most of the photoionization of bovine somatropin occurred at the glass interface of the lyophilized cake [32]. This further supports the possible hypothesis that somatropin in its lyophilized form may structurally orient in a way favoring the side with methionine 125, tryptophan, and cysteines 53/164 towards the glass of the vial. Cysteine and tryptophan oxidation were searched for in our tested samples but were not detected. The lack of oxidation of tryptophan and cysteine residues in our study supports the hypothesis by Miller et al*.* that after photoionization of tryptophan and disulfide reduction in the solid state, back electron transfer is possible to restore tryptophan and the disulfide bridge leading to no net observable oxidation byproducts [32].

Together these data show that light can induce PTMs on somatropin whether the protein is presented in a lyophilized, reconstituted, or diluted form with two methionine residues being particularly susceptible to this modification. While our findings show concentration-dependent methionine oxidation similar to previous mAb studies conducted under visible light alone [28], the combined UV/visible light exposure conditions may involve additional direct photochemical mechanisms beyond the photosensitizer-mediated pathways proposed for visible light-only degradation. Further investigation would be needed to distinguish between concentration-dependent photosensitization effects and direct UV-induced oxidation mechanisms in somatropin and other protein formulations.

## Conclusions

Collectively these data indicate that light exposure can induce product quality related changes in somatropin, whether it is in solid or liquid dosage forms. We determined via SEC that light exposure induced HMWS in all somatropin presentations with increased effect on liquid formulations. We demonstrated that light exposure induced increases in both acidic and basic charge variants via icIEF and increases in methionine oxidation via LC–MS/MS peptide mapping. However, no change in total or large particulate counts were observed via MFI. Historically, many protein-based drug products have ‘protect from light’ designations for their packaged forms but lack this instruction or designation for their reconstituted and diluted in use presentations [4]. Our data suggested that these protein presentations and in-use conditions may be susceptible to light induced quality changes.

## Supplementary Information

Below is the link to the electronic supplementary material.

ESM 1 (PDF 442 KB)

## Data Availability

The datasets generated during and/or analyzed during the current study are available from the corresponding authors on reasonable request.
