# Supplementary material for: Physicochemical Differences Observed in Photostability Studies of Lyophilized, Reconstituted, and Diluted Somatropin
Source: Pharm Res. 2025 Dec 16;43(1):185–93. doi: 10.1007/s11095-025-03986-1 (PMC12913286; doi:10.1007/s11095-025-03986-1)
Supplement: Supplementary file 1 — (PDF 442 KB) [file 11095_2025_3986_MOESM1_ESM.pdf]

Supplemental information:

Physicochemical differences observed in photostability studies of lyophilized, reconstituted, and diluted somatropin

Jordan D. Pritts[1b], Uriel Ortega-Rodriguez[1], Ashutosh Rao[2a]

Affiliations
[1]Office of Pharmaceutical Quality Research, CDER, U.S. FDA
[2]Office of Pharmaceutical Quality Assessment, CDER, U.S. FDA

Contact information
[a]Corresponding author: V. Ashutosh Rao, Ph.D., Office of Pharmaceutical Quality Assessment, Center for Drug Evaluation and Research, Food and Drug Administration, Silver Spring, MD, USA, 10903 New Hampshire Ave / Bldg. 71 Rm 2240, Silver Spring, MD, 20993
e-mail: ashutosh.rao@fda.hhs.gov; phone: 240-402-7338
[b]Co-Corresponding author: Jordan D. Pritts, Ph.D., Office of Pharmaceutical Quality Research, Center for Drug Evaluation and Research, Food and Drug Administration, Silver Spring, MD, USA, 10903 New Hampshire Ave / Bldg. 52/72 Rm 2211, Silver Spring, MD, 20993
e-mail: jordan.pritts@fda.hhs.gov; phone: 240-402-5200

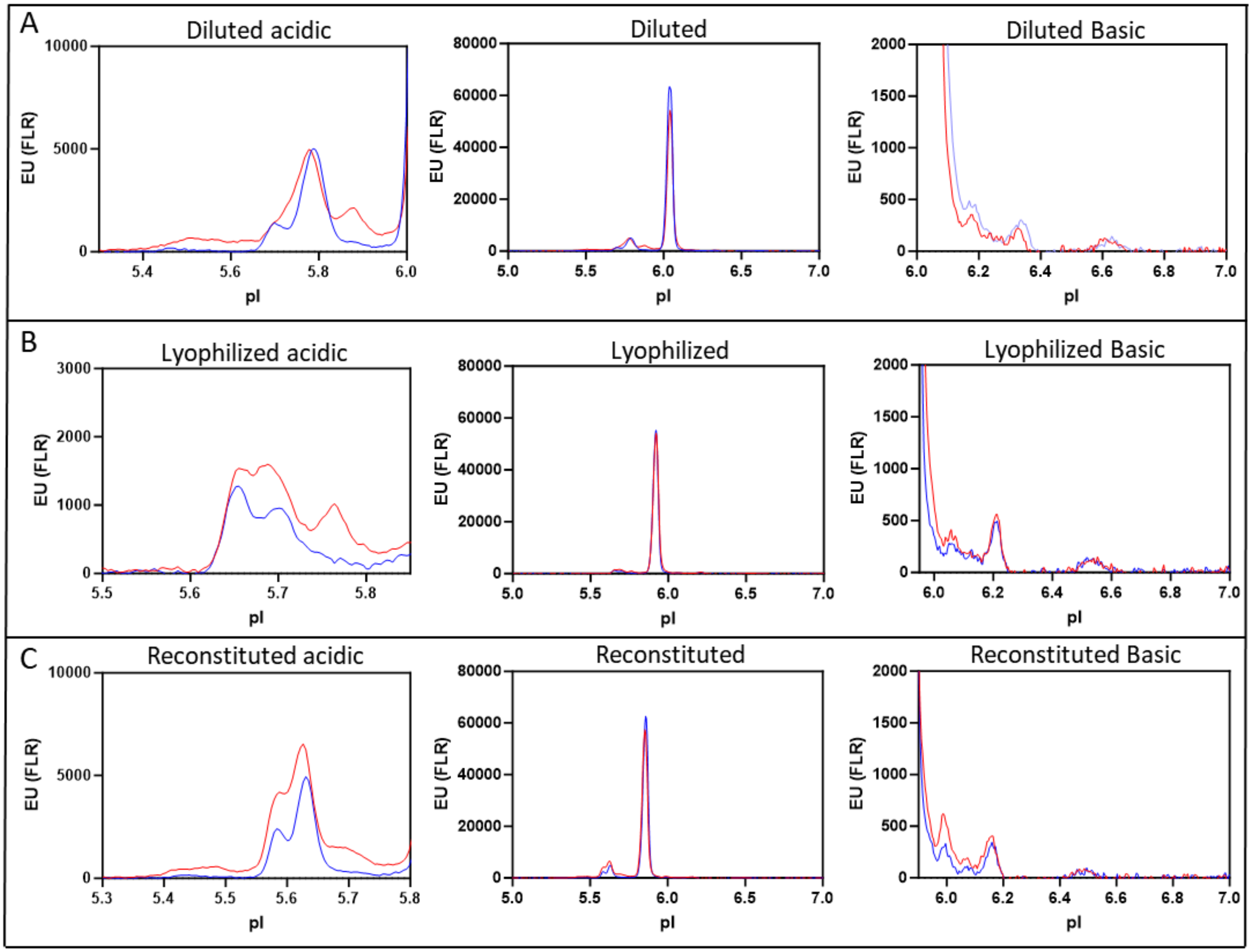

Figure S1. Analytical characterization of somatropin post light exposure via icIEF. Representative chromatograms of samples post light exposure highlighting the acidic variants (left panels), main peak (middle panels), and basic variants (right panels) sub divided by sample type including diluted (A), lyophilized (B), and reconstituted (C). Performed in technical triplicate with representative chromatograms shown. Dark controls shown in blue with light exposed samples shown in red.

Table S1. Summary of modified peptides identified by LC-MS/MS analysis.

Peptide	M Calculated (Da)	M Observed	M Observed (Da)	Delta M (Da)	ppm	Modification
LFDNAMLR	978.5029	$[490.2589]^{2+}$	978.5178	-0.0149	-15.23	M14: Unmodified
LFDNA**M**LR	994.4929	$[498.2530]^{2+}$	994.506	-0.0131	-13.17	M14: Oxidation
DLEEGIQTLMGR	1360.6729	$[681.3400]^{2+}$	1360.68	-0.0071	-5.22	M125: Unmodified
DLEEGIQTL**M**GR	1376.6629	$[689.3390]^{2+}$	1376.678	-0.0151	-10.97	M125: Oxidation
FDTNSHNDDALLK	1488.6917	$[497.2614]^{3+}$	1488.7607	-0.069	-46.35	N149orN152: Unmodified
FDT**N**SH**N**DDALLK	1489.676	$[497.5855]^{3+}$	1489.7330	-0.057	-38.26	N149orN152: Deamidation

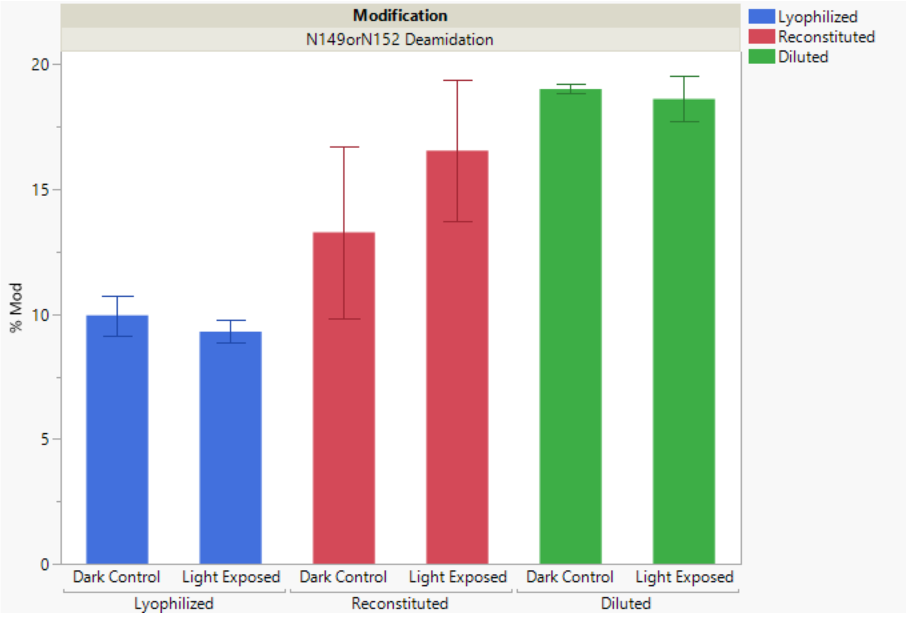

Figure S2. Deamidation analysis of somatropin post light exposure via LC-MS. Bar graphs showing % deamidation of lyophilized, reconstituted, and diluted presentations. Performed in triplicate with mean ± SD shown.

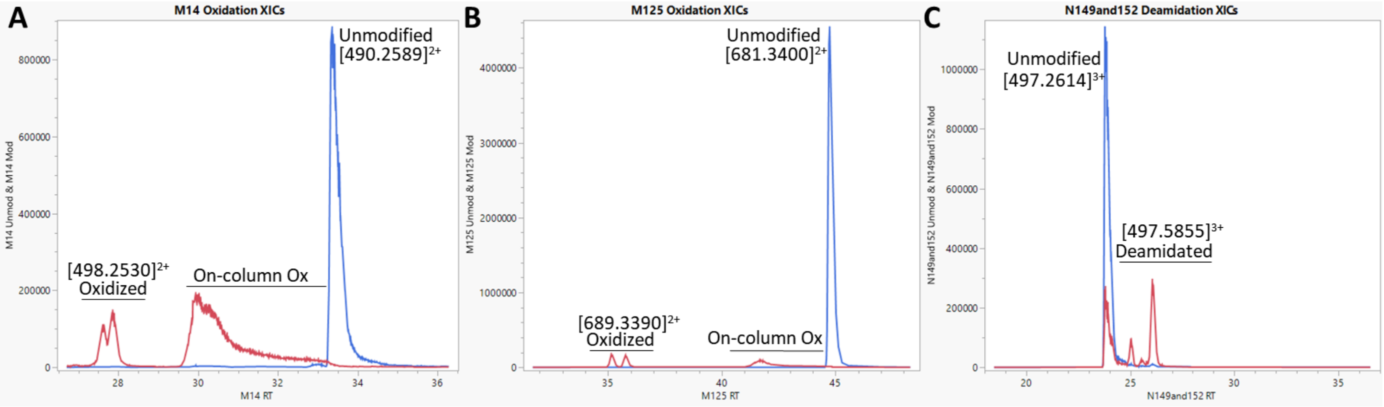

Figure S3. Overlayed representative extracted ion chromatograms (XICs) of modified and unmodified peptides (A) M14 oxidation, (B) M125 oxidation, and (C) N149or152 deamidation. Unmodified peptides shown in blue and modified peptides shown in red. Methionine oxidation XICs show on column oxidation. Peak areas of methionine on column oxidation were combined with unmodified peaks areas for subsequent PTM quantification analysis.
